# Coexistence of Eosinophilic Granulomatosis With Polyangiitis and Allergic Bronchopulmonary Aspergillosis: A Fascinating Relationship

**DOI:** 10.7759/cureus.57917

**Published:** 2024-04-09

**Authors:** Alessandro Maria Marra, Pietro Curci, Giovanni Franco, Giulia Pittalis, Erica Tugnoli, Davide Cavasin, Andrea Cristiano, Francesco Bini

**Affiliations:** 1 Pneumology Unit, Azienda Socio Sanitaria Territoriale (ASST) Rhodense, Milan, ITA; 2 Respiratory Unit, School of Medicine and Surgery, Università degli Studi di Milano Bicocca, Fondazione IRCCS San Gerardo dei Tintori, Monza, ITA

**Keywords:** il-5, mepolizumab, eosinophilic granulomatosis with polyangiitis, allergic bronchopulmonary aspergillosis, eosinophilia

## Abstract

Elevated eosinophil counts are associated with various diseases, including eosinophilic granulomatosis with polyangiitis (EGPA) and allergic bronchopulmonary aspergillosis (ABPA). EGPA is a rare small-vessel vasculitis characterized by asthma, eosinophilia, fleeting pulmonary infiltrates, and systemic manifestations. ABPA, initiated by immune reactions against *Aspergillus fumigatus* in the airways, presents with poorly controlled asthma, wheezing, hemoptysis, productive cough, and systemic symptoms, which result in characteristic central bronchiectasis. Fleeting pulmonary opacities are common radiologic findings. We present a case of ABPA in a patient with a prior EGPA diagnosis under treatment with mepolizumab 300 mg monthly and review eight similar cases from the literature. In these cases, EGPA and ABPA diagnoses preceded each other or were concurrent. Treatment of the latter improved control of both diseases. IL-5 is pivotal in EGPA pathogenesis, and mepolizumab, targeting IL-5, has been effective in EGPA treatment. Our patient received mepolizumab for EGPA and continued it post-ABPA diagnosis, showing favorable outcomes. This suggests mepolizumab as a therapeutic link between EGPA and ABPA. Mepolizumab therapy holds promise for managing both EGPA and ABPA. Double-blind placebo-controlled studies are warranted to establish its efficacy and safety for ABPA, emphasizing the need for further research in this area.

## Introduction

Elevated eosinophil counts play a significant role in various diseases, spanning from more prevalent organ-specific disorders like severe eosinophilic asthma to rarer multisystem conditions such as eosinophilic granulomatosis with polyangiitis (EGPA) and hypereosinophilic syndrome (HES). Patients with these multisystem disorders are at considerable risk of morbidity and mortality due to delays in diagnosis or insufficient treatment [1]. EGPA is a rare small-vessel vasculitis that occurs in patients with asthma and eosinophilia histologically characterized by tissue eosinophilia, necrotizing vasculitis, and eosinophil-rich granulomatous inflammation [2]. EGPA typically progresses through three distinct phases: a prodromal phase marked by asthma and chronic rhinosinusitis, an eosinophilic phase during which eosinophilia and organ involvement emerge, and a vasculitic phase characterized by clinical manifestations due to small-vessel vasculitis [3].

Asthma is a prominent aspect of EGPA, typically occurring before the onset of vasculitis symptoms (mean duration of 9.3 ± 10.8 years) [4]. It affects 90-100% of patients and exhibits distinct characteristics compared to asthma in the general population. Asthma in EGPA is typically of late onset and severe in nature and frequently necessitates long-term oral corticosteroid (OCS) treatment, even after the systemic disease has regressed [5]. An allergic background is present in less than one-third of patients with EGPA. Atopy, if present, is associated with a better prognosis but with more severe or uncontrolled asthma manifestations in the year before the development of vasculitis.

Lung involvement is present in 37-98% of patients with EGPA, depending on the study series. In addition, a chest radiograph is abnormal in 70% of patients and shows bilateral pulmonary consolidative or reticulonodular opacities in a peripheral distribution. In high-resolution computed tomography (HRCT), which is a precise method, pulmonary lesions can be classified as airspace and airway patterns [6]. 

Allergic bronchopulmonary aspergillosis (ABPA) is an inflammatory disease caused by immunologic reactions initiated against *Aspergillus fumigatus* colonizing the airways of patients with asthma and cystic fibrosis [7]. Classic ABPA pathogenesis involves fungal sensitization to *Aspergillus *species, which act as a growth factor for eosinophils, potentiating effects of IL-3, IL-5, and growth colony-stimulating factor. Eosinophils interact directly with *A. fumigatus* spores to generate eosinophilic extracellular traps, which can injure bronchial epithelium. Clinical manifestations of ABPA include poorly controlled asthma, wheezing, hemoptysis, productive cough, and systemic symptoms, such as fever and weight loss, which result in characteristic central bronchiectasis. Fleeting pulmonary opacities are common radiologic findings [8]. New diagnostic criteria for patients without cystic fibrosis include meeting at least six of the following criteria: history of asthma symptoms, peripheral blood eosinophilia (≥500 cells/μL), elevated total serum IgE (≥417 IU/mL), immediate cutaneous hypersensitivity or specific IgE for filamentous fungi, presence of precipitins or specific IgG to filamentous fungi, filamentous fungal growth in sputum cultures or bronchoalveolar lavage (BAL), central bronchiectasis on computed tomography (CT) scan, presence of mucus plugs in the central bronchi or history of mucus plug expectoration, and/or high attenuation mucus in the bronchi on CT [9].

EGPA and ABPA enter into the differential diagnosis when we are faced with a lung disease with eosinophilia. The coexistence of EGPA and ABPA is extremely rare, but overlapping features between the two are described. Differentiation between EGPA, ABPA, and asthma exacerbation is crucial in directing subsequent management. We report a case of ABPA in a patient with a previous diagnosis of EGPA and compare it with other similar cases in the literature. This is the only case we know of in the literature where a patient has undergone treatment with mepolizumab for EGPA and continued it even during ABPA.

The aim of this case-based review is to describe the relationship between these two pathologies, considering the few cases in which they coexist in the same individual in the literature, identifying the anti-IL-5 monoclonal antibody mepolizumab as a possible treatment that might be beneficial for both conditions.

## Case presentation

**Figure 1:**
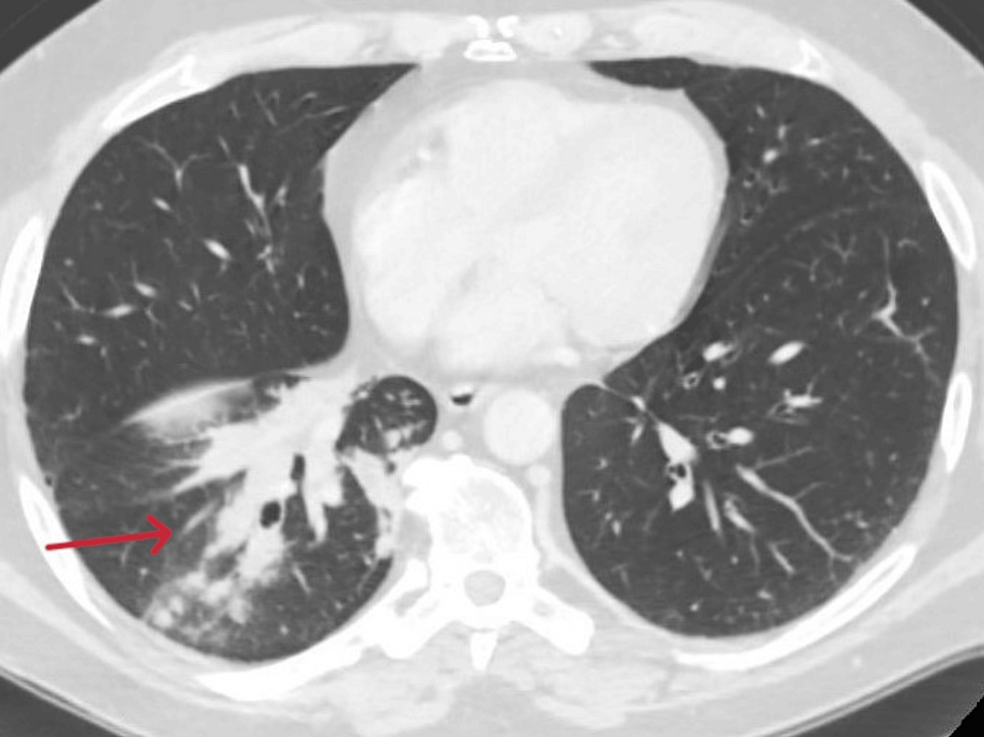
CT scan of the patient at diagnosis of EGPA: bronchiectasis associated with ground-glass pulmonary infiltrates CT: computed tomography, EGPA: eosinophilic granulomatosis with polyangiitis

A 62-year-old Caucasian man presented with worsening dyspnea, cough, and a significant medical history including asthma with frequent exacerbations, allergic rhinitis, nasal polyposis, and previous intestinal polyposis with eosinophilic component on histological examination. In addition, the patient experienced numbness and loss of strength in all four limbs, along with blood hypereosinophilia with eosinophils measuring 1.8 x 109/L (21%) and weakly positive P-ANCA (7 IU/mL). A CT scan revealed bronchiectasis associated with ground-glass pulmonary infiltrates (Figure 1), while an electroneurography indicated sensorimotor axonal neuropathy with asymmetric distribution (mononeuritis multiplex). Transbronchial and muscular biopsies showed vasculitis with extravascular eosinophils.

A diagnosis of EGPA was made, and treatment commenced with prednisone at 1 mg/kg with subsequent tapering, followed by monthly mepolizumab 300 mg, resulting in good disease control. Despite therapy, a follow-up CT scan after three months still revealed pulmonary infiltrates, with the patient reporting only a persistent cough and no asthma exacerbations or other symptoms. Videobronchoscopy revealed hyperemic and easily bleeding airway mucosa with whitish secretions, prompting further investigation with diffuse bronchus aspirate, medium bronchoalveolar lavage, and multiple bronchial and transbronchial biopsies. Bacterial, fungal, and mycobacterial cultures were negative, but bronchial cytogram showed eosinophils, and histologic examination demonstrated fragments of lung parenchyma with occasional hyperplastic pneumocytes and very rare eosinophilic granulocytes.

**Figure 2:**
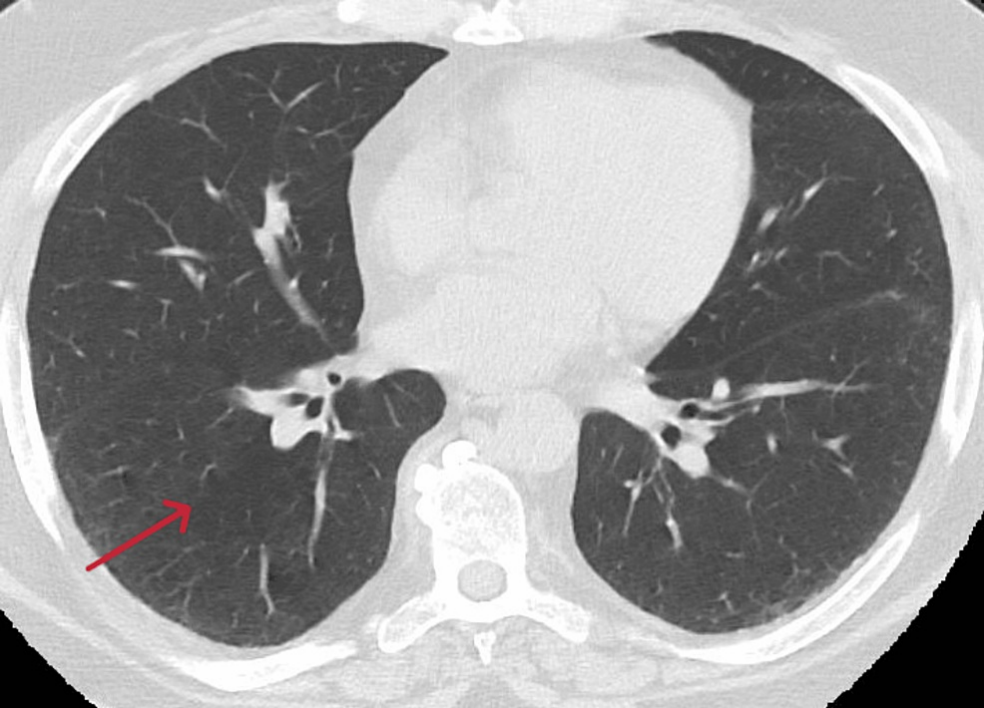
CT scan of the patient six months after treatment for ABPA: no pulmonary infiltrates CT: computed tomography, ABPA: allergic bronchopulmonary aspergillosis

Further laboratory tests revealed normal eosinophil levels, total IgE of 2484 IU/mL, and positive specific IgE for *A. fumigatus *(68.7 kU/L), recombinant Asp f 4, and Asp f 6, along with positive IgG for *A. fumigatus*. Based on the new 2021 diagnostic criteria, the patient was diagnosed with ABPA and continued therapy with mepolizumab and prednisone. Isavuconazole was added for four months and then stopped. A subsequent CT scan after six months showed no pulmonary infiltrates (Figure 2), leading to the tapering and discontinuation of OCS therapy, which was not restarted thereafter. The patient continued therapy with mepolizumab 300 mg with good control of both conditions. This is the first case within our knowledge where both these conditions are present in a patient treated with anti-IL-5 biological therapy. After antifungal treatment, the patient continued maintenance therapy with mepolizumab, maintaining remission of EGPA without the emergence of new symptoms.

## Discussion

A literature search was conducted in January 2024 using the PubMed search engine (PubMed, National Library of Medicine, Bethesda, MD) to access the MEDLINE and Scopus databases. The search query included keywords, such as “eosinophilia,” “allergic bronchopulmonary aspergillosis (ABPA),” “eosinophilic granulomatosis with polyangiitis (EGPA),” “IL-5,” and “mepolizumab.” The search was limited to articles published over the last 15 years, with some older papers of particular interest also included. Reports older than 2009 and reports with incomplete data have been excluded. A narrative case-based review was conducted, considering relevant literature, including original articles and reviews. In addition, the reference lists of all articles were scanned to identify any references not initially found in the research. Eight cases of coexisting EGPA and ABPA were included in this paper, bringing the total number of cases reviewed to nine (Table 1).

**Table 1:** Patients with EGPA and ABPA: main features of the cases reviewed EGPA: eosinophilic granulomatosis with polyangiitis, ABPA: allergic bronchopulmonary aspergillosis, p-ANCA MPO: perinuclear antineutrophil cytoplasmic antibody-myeloperoxidase

Author	Gender	Age at first diagnosis	First diagnosis	Second diagnosis	Eosinophil count	IgE level	Autoantibodies	Treatment for EGPA	Antifungal agent for ABPA	Outcome
Lee W et al. [10]	M	61	EGPA	ABPA, one year later	22.9 x 10^9^/L	7610 IU/mL	None	a) First line for EGPA: prednisolone, then added cyclophosphamide 50 mg/daily for a year. b) Manteinance for EGPA: azathioprine (stopped at diagnosis of ABPA). c) For ABPA: prednisolone 0.5 mg/kg/daily for three weeks, then tapered to 7.5-10 mg/daily	Itraconazole	a) EGPA: clinical remission. b) ABPA: improvement of symptoms and laboratory markers
											Ren S [11]	F	41	Concomitant diagnosis		1.6 x 10^9^/L	1326 kU/L	p-ANCA MPO	Prednisone 35 mg/die, then added azathioprine 1.5 mg/kg/daily	Itraconazole 200 mg twice daily	a) Improvement of symptoms and radiologic signs. b) Eosinophil normalization and pANCA negativization. c) Prednisone tapering
																						Ishiguro T et al. [12]	M	47	EGPA	ABPA, 6 years later	4.4 x 10^9^/L	1115 IU/mL	None	Prednisolone 40 mg/daily	None	a) Eosinophilic pneumonia and polyneuropathy on admission, then improvement of symptoms and steroid tapering. b) Eosinophilic cardiomyositis and heart failure after six years, retrospective diagnosis of EGPA. c) Pacemaker implantation. d) Prednisolone increase
F	47	EGPA	ABPA, 2 years later	12.2 x 10^9^/L	1205 IU/mL	p-ANCA MPO	Prednisolone 40 mg/daily	None	a) Improvement of symptoms and radiologic signs. b) Steroid tapering. c) Alive after 11 years from diagnosis of EGPA																							
										M	58	ABPA	EGPA, 6 years later	1 x 10^9^/L	Increased (value not specified)	p-ANCA MPO	Prednisolone 30 mg/daily	None	a) Prednisolone 30 mg/daily started at ABPA diagnosis, then tapered to 5 mg/daily. b) Six years later, developed of fever, myalgia, sinusitis, otitis media, and diagnosis of EGPA. c) Prednisolone 30 mg/die again, improvement of hearing. d) Alive after one year follow-up													
																				Henderson SR et al. [13]	M												72	ABPA	EGPA, 5 months later	7.1 x 10^9^/L	219 kU/L	p-ANCA MPO	Prednisolone 60 mg/daily + azathioprine 100 mg/daily	None	a) Improvement of symptoms and laboratory markers. b) Good clinical control with low-dose prednisolone and azathioprine and high-dose ICS
Harada M et al. [14]	M	84	Concomitant diagnosis		1.4 x 10^9^/L	1510 IU/mL	p-ANCA MPO	Prednisolone 0.5 mg/kg/daily	Itraconazole 200 mg/daily														a) Improvement of symptoms and radiologic signs. b) Prednisolone tapered to 15 mg/daily																		
																				Alen Coutinho I et al. [15]	M			27	Concomitant diagnosis		1.5 x 10^9^/L	5175 IU/mL	p-ANCA MPO	Methylprednisolone 1 mg/kg/daily	Voriconazole, then liposomal amphotericin B, then itraconazole	a) Resolution of symptoms. b) Itraconazole suspended after 20 days. c) Corticosteroids tapered until stop
Marra AM et al.	M	62	EGPA	ABPA, 3 months later	1.8 x 10^9^/L	2484 IU/mL	p-ANCA MPO	Prednisone 1 mg/kg/die, mepolizumab 300 mg monthly	Isavuconazole	a) Improvement of symptoms. b) Radiological remission after six months of antifungal treatment																						

Instances of concurrent EGPA and ABPA have been reported in the literature [10-15]. EGPA may precede or follow ABPA, and they can be diagnosed concurrently. Among the cases included in our review, five were isolated case reports. One author reported a small review of four cases in 2012 [11], while another reported three cases in a larger review of six cases of ABPA associated with EGPA [12], mostly from articles written in Japanese and predating 2009. Of the nine cases of EGPA and ABPA reported to date (including ours), seven were males and two were females with a mean age at onset of the first pathology of 55.4 ± 17.1 years (range 27-84 years). The clinical characteristics of the patients are summarized in Table 1.

In four cases, including ours, the diagnosis of EGPA was made first, with ABPA later appearing or being recognized. In three cases, the diagnoses were concurrent. In two other cases, ABPA was diagnosed first: In one case, EGPA manifested after six years with constitutional symptoms not present at the time of ABPA diagnosis. In another case, EGPA manifested after six months from the diagnosis of ABPA without specific antifungal therapy except for OCS therapy. All nine patients were treated with OCSs at diagnosis, while only five out of nine immediately underwent antifungal therapy for ABPA. In four of the cases reviewed, the therapy for ABPA primarily consisted of higher-dose steroid therapy. The patient examined in our case is the only one who underwent biological therapy with mepolizumab, which was continued even after the diagnosis of ABPA. In all cases, the outcomes for both conditions improved with treatment of the last diagnosed disease. Patients who underwent antifungal therapy for ABPA more easily achieved improvement for EGPA as well. We reviewed eight cases of coexisting EGPA and ABPA from the literature, in addition to our case. In all cases, the treatment of the latter improves control of both diseases. Steroid and antifungal treatment allows for a rapid improvement in the signs and symptoms of ABPA, and consequently, this also improves outcomes for EGPA.

The relationship between ABPA and EGPA is not completely understood. Some diagnostic criteria are common, and differential diagnosis is recommended. Several reports described the sequential occurrence of ABPA and EGPA, or vice versa [10-15]. How one established disease may predispose to the other is unknown, but it may include eosinophil recruitment by T helper 2 (Th2)-driven inflammation predisposing to fungal colonization and subsequent hypersensitivity [15].

In 2020, authors from Japan proposed a concept of “adult-onset eosinophilic airway diseases,” including asthma, ABPA, EGPA, and other eosinophil-driven diseases. Common pathophysiology and inflammation pathways seem to underlie them. In addition, each disease exhibits a specific pathophysiology, such as fungal sensitization in ABPA and autoimmunity in EGPA [16].

Several cell types participate in the immunopathogenesis of EGPA. Eosinophils are clearly central and are likely to mediate tissue damage, a concept supported by the evidence that targeting IL-5 (e.g., using mepolizumab), a survival factor for eosinophils, is an effective therapy for EGPA [17]. In our case, the patient started treatment with mepolizumab for EGPA according to recommendations for the treatment of EGPA published in 2021 [18] as soon as possible and did not discontinue it after the subsequent diagnosis of ABPA. This is the first case within our knowledge where both these conditions are present in a patient treated with anti-IL-5 biological therapy. In all cases reviewed, the outcomes for both conditions improved with treatment of the last diagnosed disease. Patients who underwent antifungal therapy for ABPA more easily achieved improvement for EGPA as well. Antifungal treatment is essential to improve the outcomes of ABPA, as described in various scientific studies [7-8]; however, considering and treating the IL-5 pathway might prove to be a winning choice.

Regarding the IL-5 pathway, mepolizumab has been effectively used in patients with severe refractory eosinophilic asthma and has been introduced as an add-on therapy in EGPA, with good results [19]. Data on the safety and impact of mepolizumab treatment in clinical trials and real-world settings demonstrate its clinical benefits across the eosinophil-driven disease spectrum (EGPA, severe eosinophilic asthma, chronic rhinosinusitis with nasal polyps, and HES), providing valuable insights into the importance of eosinophils in these chronic inflammatory diseases [20].

Based on the role of IL-5, a marked eosinophilia in peripheral blood and bronchial washing suggests that beneficial effects may result from treatment with mepolizumab in ABPA, as reported in the literature [21-23]. ABPA pathogenesis involves fungal sensitization to *Aspergillus* species, which act as a growth factor for eosinophils, potentiating the effects of IL-3, IL-5, and the growth colony-stimulating factor. Eosinophils interact directly with *A. fumigatus* spores to generate eosinophilic extracellular traps, which can injure bronchial epithelium. Pulmonary eosinophilia is a hallmark of ABPA. Based on this, the International Society for Human and Animal Mycology (ISHAM) working group for ABPA has suggested a cutoff of ≥500 cells/μL for peripheral blood eosinophil count for the diagnosis of ABPA [9]. Recently, it was shown that *A. fumigatus* induces the formation of eosinophilic extracellular traps that lack the killing or fungistatic activities against *A. fumigatus* and contribute to the pathology of ABPA [24].

The innate immune response to *A. fumigatus* in the lung leads to chemotaxis and activation of Th2 cells. Th2 cells produce cytokines, including IL-4 and IL-5 that drive the differentiation of IgE-secreting plasmacytes and attract and activate eosinophils [21]. Our literature findings have shown that antifungal treatment allows for a rapid improvement in the signs and symptoms of ABPA, and consequently, this also improves outcomes for EGPA.

In the case of our patient, the diagnosis of ABPA was based on radiological findings rather than disease symptoms, which were mild and well-controlled; thus, he continued mepolizumab 300 mg, and after antifungal treatment, he controlled EGPA with the disappearance of ABPA lung infiltrates after six months with maintenance of remission of both conditions. Our case suggests the positive outcome of mepolizumab treatment in both diseases. Therapies targeting IL-5, such as mepolizumab, hold promise in managing both EGPA and ABPA, indicating a therapeutic connection between the two conditions.

## Conclusions

The coexistence of EGPA and ABPA in the same patient is very rare but possible, and the relationship between the two is fascinating. It is not yet clear what mechanisms may promote one over the other, but eosinophilia and IL-5 likely play a role. Mepolizumab might be considered a therapeutic link between EGPA and ABPA. This finding represents a significant advancement in understanding the pathogenesis of these disorders, underscoring the need for further research in this area. It is noteworthy that double-blind placebo-controlled studies are required to establish the efficacy and safety of mepolizumab therapy for ABPA. Further studies are needed to support the efficacy of mepolizumab in ABPA in large trials.
